# Ketogenic diet for alternating hemiplegia of childhood: Case report and literature review

**DOI:** 10.1097/MD.0000000000044993

**Published:** 2025-10-03

**Authors:** Yu Yang, Penghui Liu, Pei Li, Yanru Huang, Shuxiang Hu

**Affiliations:** a Department of Pediatric Neurological Rehabilitation, Department of Pediatrics, Women and Children’s Hospital, School of Medicine, Xiamen University, Xiamen, Fujian, China; b The First Clinical Medical College of Fujian Medical University, Fuzhou, Fujian, China; c Department of Central Laboratory, Fujian Key Clinical Specialty of Laboratory Medicine, Women and Children’s Hospital, School of Medicine, Xiamen University, Xiamen, Fujian, China.

**Keywords:** alternating hemiplegia of childhood, *ATP1A3*, ketogenic diet

## Abstract

**Rationale:**

Alternating hemiplegia of childhood (AHC) is a serious and rare neurological disease caused by mutations in the *ATP1A3* gene. Patients mainly present with paroxysmal hemiplegia, dystonia, abnormal eye movement, dyspnea, and other autonomic neurological phenomena.

**Patient concerns:**

Here, we described a patient who initially presented with abnormal eye movements, followed by the subsequent development of seizures and alternating hemiplegia.

**Diagnoses:**

Whole exome sequencing identified a heterozygous variant in the *ATP1A3* gene: c.2443G > A (p.Glu815Lys). Thus, the patient was diagnosed with AHC.

**Interventions:**

Flunarizine combined with oxcarbazepine did not improve clinical symptoms.

**Outcomes:**

Interestingly, typical AHC paroxysmal episodes gradually improved after starting the ketogenic diet, but the seizures did not resolve. Long-term follow-up shows a global improvement in neurological development.

**Lessons:**

Our case reinforces the role of the ketogenic diet as a novel therapeutic option for AHC. However, further specific confirmatory tests are necessary.

## 1. Introduction

Alternating hemiplegia of childhood (AHC) is a serious and rare neurological disorder first described in 1971.^[1]^ The main pathogenic gene of AHC was found to be *ATP1A3* in 2012, and most of the mutations were confirmed to be de novo.^[2]^,Mikati^[3]^ proposed revised diagnostic opinions on AHC in 2021. There is no effective treatment for AHC to date. In clinical practice, removing the stimulation, preventive drug treatment, treatment during seizures, and control of epileptic seizures are mainly adopted. This article reviews and analyzes the clinical data of a child with AHC treated using the ketogenic diet (KD) and includes a review of domestic and foreign literature.

## 2. Case report

We report a 4 years and 10 months old boy with an unremarkable family history, who was admitted to our hospital due to “episodic eye movement abnormalities, hemiplegia, and convulsions for more than 4 years.” He was the first child of healthy, unrelated parents. The pregnancy period of mother and neonatal period of the child were normal. Abnormal eye movements first occurred at 2 months, manifesting as eyeball upturning or staring lasting for several minutes. Video EEG revealed the absence of an epileptic wave during the occurrence of abnormal eye movements. Whereafter, the patient was diagnosed with epilepsy. Initial treatment with oral levetiracetam liquid proved to be ineffective, the parents discontinued administering the drug after one and a half months. At the age of 5 months, the patient experienced their first convulsive seizure, characterized by bilateral tonic‐clonic seizures lasting for up to 30 minutes. The patient’s parents, worried about the side effects of the drugs, refused to take anti-seizure medications (ASM). The mean seizure frequency was about 3 to 5 per year at later times, still presenting as bilateral tonic‐clonic seizures frequently with status epilepticus. Episodic paralysis of the limbs occurred at the age of 9 months, lasting from 10 minutes to more than 20 hours each time. Paralysis occurred on the left or right side and sometimes multiple times a day in alternate limbs without simultaneous paralysis of all 4 limbs. The hemiparesis had no obvious regularity, was easily triggered by bathing, strong light, and strong sounds, improved after fell asleep and recovered after woke up. At the age of 3 years and 11 months, the child improved the examination of whole exome sequencing and confirmed the diagnosis of AHC. Afterwards, flunarizine was administered orally, initially at 2.5 mg once a day and increasing to 5 mg once a day after half a month, oxcarbazepine was added for the control of seizures. However, abnormal eye movement and hemiplegia did not decrease, the control of seizures was not ideal, so the parents stopped using oxcarbazepine 9 months later.

**Figure 1.**
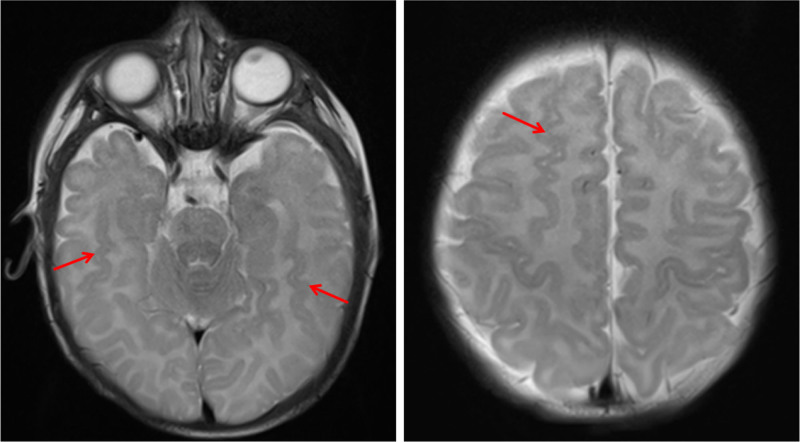
The brain MRI revealed gray matter heterotopia in the bilateral frontotemporal regions. The arrow points to the ectopic area of gray matter.

**Figure 2.**
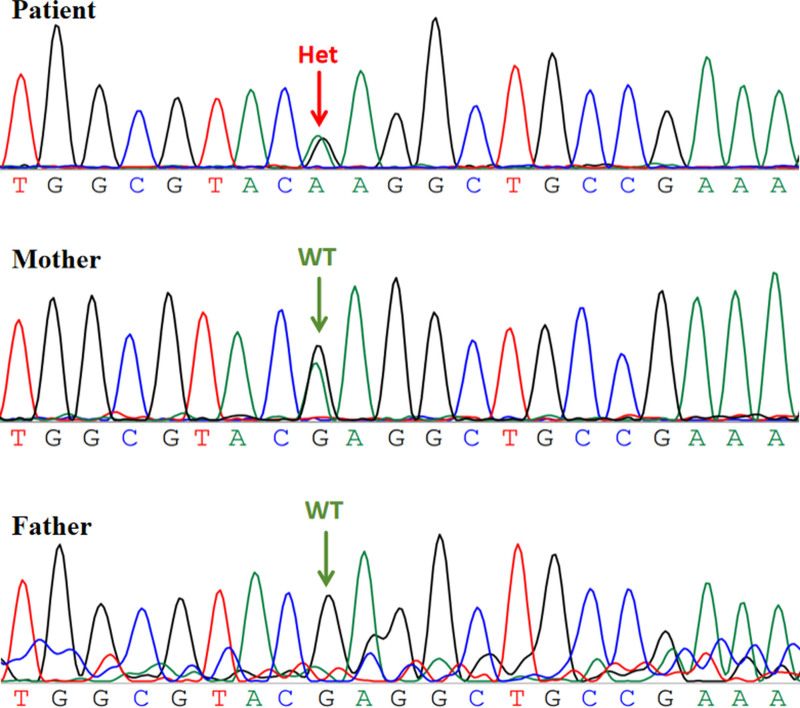
Sanger sequencing of target genes. The red arrow indicates the *ATP1A3* gene c.2443G > A (p.Glu815Lys) mutation in the patient. Green arrows indicate sequencing results from normal members without mutation in the parents.

The patient presented to our hospital at the age of 4 years and 10 months. He was still unsteady, showed frequent abnormal eye movements, the hemiplegic attacks lasted from 10 minutes to 24 hours and occurred about 7 to 30 times a week, seizure frequency varied from 3 to 5 seizures per year, Psychomotor development of this patient was significantly backward compared with children of the same age. He could not stand or walk alone and consciously speak at this point. Ancillary examinations revealed the following: fundus examination and brainstem evoked potentials showed no abnormality. Blood biochemistry, ammonia, lactate, tandem mass spectrometry, and urine gas mass spectrometry, routine chromosome tests were all normal. Six video EEGs obtained from 2 months to 4 years of age were also normal. But, the brain MRI revealed gray matter heterotopia in the bilateral frontotemporal regions (Fig. 1). And whole exome sequencing identified a heterozygous variant in the *ATP1A3* gene (NM_152296.4): c.2443G > A (p.Glu815Lys) (Fig. 2).

In our hospital, considering previous treatment was not ideal for controlling hemiplegic attacks, abnormal eye movements and seizures, and parents refused to change ASM, he was started on a 2:1 KD after obtaining informed consent from the parents. Ketogenic ratios subsequently increased to 2.5:1, 3:1, 4:1 on the basis of tolerance. During the diet, parents recorded the paroxysmal episodes and adverse reactions daily by event calendar. Blood lipids and blood glucose levels were monitored before KD treatment, after the first month, and every 3 months thereafter. We had a weekly follow-up for the first month, then follow-up once a month for 1 year. Gesell Developmental Schedules were performed before KD and 6, 12 months after the diet by a single professional experimenter who was blind to the treatment.

**Figure 3.**
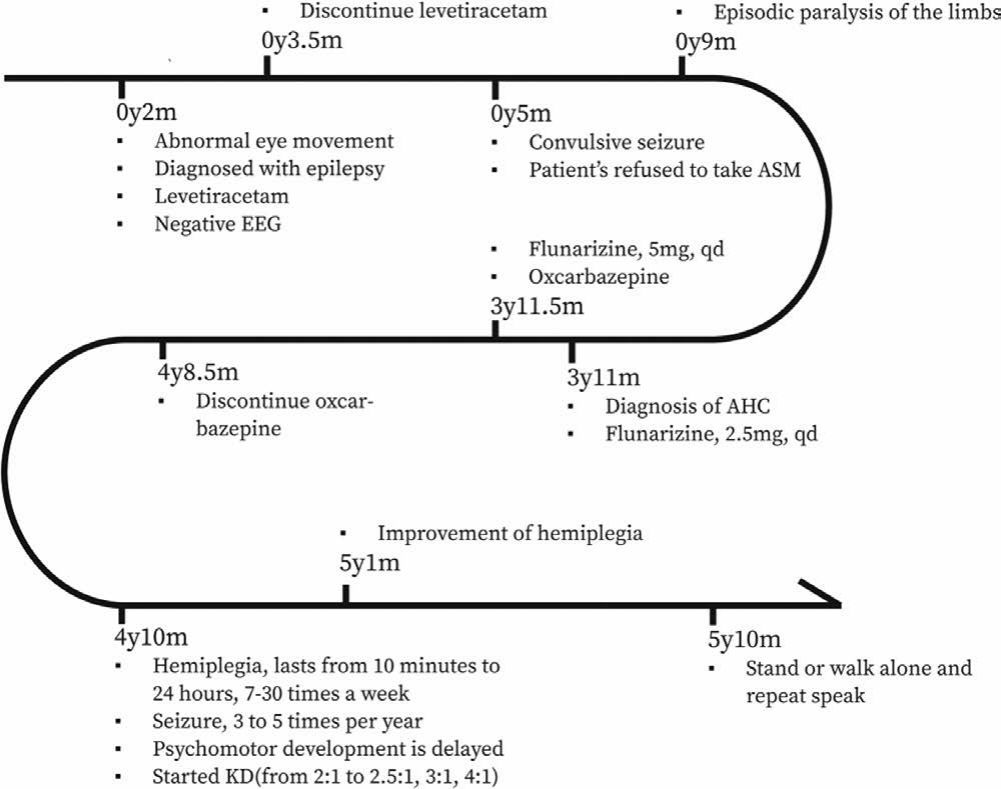
The clinical course diagram of the child patient.

Three months after initiating the KD, we were pleasantly surprised to observe an improvement in the frequency and severity of hemiplegia. This improvement continued after 6 and 12 months of following the diet, with the symptoms of hemiplegia becoming milder, shorter, and less frequent. Although the parents did not keep precise records of abnormal eye movements, they subjectively noticed a significant reduction in them. Additionally, there was a notable improvement in neuropsychological development across all areas. By following the diet for 12 months, the patient progressed from sitting unsteadily to sitting alone and standing up, from babbling to repeating words such as “sister” and “grandma,” and from grabbing biscuits to eating them (Fig. 3). Unfortunately, the frequency and severity of seizures had not been in remission. Table 1 presents the observation indexes of the clinical manifestations before and after KD treatment.

**Table 1:** Clinical observations of the patient before and after ketogenic diet treatment.

Observation indicator		Before ketogenic therapy	Ketogenic diet for 6 months	Ketogenic diet for 12 months
Seizures		3–5 times/yr	3 times in 6 mo	5 times in 12 mo
Hemiplegia		1 to several times a day, lasts longer than 24 h	At least once every other day, lasts up to 8 h	At least once every other day, lasts up to 3 h
Gesell (DQ)	Gross movement	10	12	15
	Fine motor	6	10	13
	Socially adaptive behavior	6	10	15
	Language	6	13	16
	individual-society	6	13	14
	Total DQ	7	12	15

DQ = developmental quotient.

## 3. Discussion

AHC is a rare neurological disease characterized by paroxysmal episodes of hemiplegia, dystonia, abnormal eye movements, dyspnea, and other autonomic phenomena with onset before 18 months of age, whereas symptoms are usually relieved by sleepl.^[3]^ In 2012, Heinzen reported for the first time that the *ATP1A3* gene was the main pathogenic gene responsible for AHC, and most of the mutations were de novo.^[2]^ More than 80 kinds of *ATP1A3* gene mutations have been discovered thus far, mainly missense mutations. A few are splicing mutations and base-deletion frameshift mutations. The 2 most common mutations are c.2401G > A (p.Asp801Asn) and c.2443G > A (p.Glu815Lys), with the former accounting for 40.0% and the latter accounting for 24.2%.^[2]^

There is currently no specific treatment for AHC. During an attack, the duration of paralysis can be shortened by administering sedative drugs to increase sleep time. During the period between attacks, the patient has to avoid triggers for the attack, such as high or low temperatures, crowded areas, specific odors, irregular sleep, bathing, etc. Other recommendations include long-term drug treatment, with flunarizine being the most commonly used drug. The mechanism of action is still unclear but may be related to the drug’s ability to block calcium ion channels. It has been reported that the frequency, severity, and duration of hemiplegic attacks improved after flunarizine treatment in more than half of the patients.^[4]^ Topiramate can be used as a backup drug for ineffective flunarizine treatment, either alone or in combination. Although long-term use of flunarizine can reduce the frequency and duration of hemiplegic episodes and improve the quality of life in children,^[4]^ it fails to achieve seizure control.^[5]^ In addition, whether based on retrospective or prospective studies, flunarizine does not seem to improve the long-term developmental outcomes.^[6]^

The patient in our study presented with paroxysmal abnormal eye movements at the age of 2 months, epileptic seizures started at 5 months, and alternating limb hemiplegia gradually appeared at 9 months, associated with developmental delay. The diagnosis and treatment of the child was not smooth from our previous description. Whole exome sequencing at 3 years and 11 months showed a c.2443G > A (p.Glu815Lys) heterozygous variant of the *ATP1A3* gene. Thus, the patient was diagnosed with AHC. Flunarizine combined with oxcarbazepine did not improve clinical symptoms. We empirically chose KD therapy as the difficulty of convulsion control and poor compliance with ASM.

KD is a high-fat, low-carbohydrate, moderate-protein diet that simulates the metabolic response of the body under fasting conditions, using ketone bodies to replace glucose as the main energy substance in the brain. The diet has been used since the 20th century. Since its official introduction, it has become a commonly used adjuvant therapy for drug-resistant epilepsy.^[7]^ A Japanese study analyzed brain glucose metabolism by 18Ffluorodeoxyglucose PET in 5 patients with AHC.^[8]^ It was suggested that low glucose metabolism was found in the frontal lobes with all patients, and in the ipsilateral putamen with 3 patients. KD provides ketone bodies as an alternative source of energy for the brain, was shown to effectively improve the onset of all glucose transporter 1 deficiency syndrome (GLUT1-DS) symptoms: epilepsy, movement disorders, and cognitive impairment.^[9]^ It is considered to be potentially effective in the treatment of AHC as the similarity of clinical symptoms and glucose metabolism in the brain between AHC and GLUT1-DS. Interestingly, the first report on the effect of KD in a patient with AHC is accidental, the proband was misdiagnosed with GLUT1-DS at 3.5 years old. KD therapy was started, and the episodic symptoms disappeared completely after 15 months.^[10]^ A cohort study conducted in Southern Europe yielded findings indicating that 3 of 10 patients with AHC experienced notable clinical improvements when following the diet.^[11]^ Notably, 1 child who began a KD at the age of 11 and adhered to it for a year exhibited no clinical effects from flunarizine, but demonstrated significant enhancements in behavior and sociability. Likewise, the KD led to complete cessation of hemiplegic episodes and cognitive function improvement in the other 2 children who had poor responses to flunarizine or benzodiazepines. Collectively, these observations indicate that the KD holds potential as a viable therapeutic approach for AHC.

In our study, a KD was administered to the patient over a period of 12 months. The implementation of this dietary intervention resulted in a reduction in the onset of hemiplegia, a shortened duration of the condition, and a notable improvement in neuropsychological development. These findings align with previous case reports documenting the positive effects of the diet in patients with AHC. Nevertheless, it is noteworthy to mention that no significant alteration in seizure frequency was observed throughout the course of our study. Recurrent seizures are believed to be associated with ectopic gray matter. Based on a report from Italy pertaining to a case of *ATP1A3*-related epileptic encephalopathy, it was observed that the implementation of KD yielded substantial improvements in both hemiplegic episodes and overall seizure control.^[12]^ Additionally, long-term follow-up revealed noteworthy advancements in neurocognitive and motor abilities among the affected children. A subsequent examination of the therapeutic response in a cohort of 7 patients diagnosed with *ATP1A3*-associated epilepsy revealed that 4 individuals were introduced to the KD.^[13]^ Among these, 2 patients experienced a notable decrease in seizure frequency subsequent to the implementation of the diet, whereas the remaining 2 patients did not observe any reduction in seizure occurrence. It is crucial to acknowledge that further research is necessary to determine the effectiveness of the KD in controlling seizures in individuals with AHC. Table 2 compares the literature reviews with our case report.

**Table 2:** Clinical and therapeutic comparison between literature cohort and current case report.

Feature	Vila-Pueyo *et al* (2014)^[11]^ (Southern European Cohort, n = 10)	Current case report	Key comparisons
Age at onset	0–18 mo (median: 6 mo)	2 mo (ocular symptoms), 9 mo (hemiplegia)	Consistent with AHC diagnostic criteria (onset < 18 mo)
Sex distribution	Male:5, Female:5	Male	No sex predilection established
Core symptoms	Alternating hemiplegia (100%), dystonia (80%), ocular motor, abnormalities (60%) Ataxia (50%)	Alternating hemiplegia Oculomotor abnormalities Epileptic seizures	Hemiplegia/ocular abnormalities universal; epilepsy more prominent in current case
Epilepsy	3/10 (30%)	Present (onset 5 months; 3–5 seizures/yr)	Refractory seizures in current case associated with gray matter heterotopia
Hemiplegia characteristics	Unilateral/bilateral, duration: minutes–days, Sleep-responsive	Unilateral alternating, duration: 10 min–24 h, sleep-responsive	Stress-triggered and sleep resolution in both
Neuroimaging	Normal MRI: 9/10, subtle white matter lesions: 1/10	Bilateral frontotemporal gray matter heterotopia	Current case shows structural malformation (novel finding)
Genetic variant	*ATP1A3*: 5/10 (50%), Common variants: p.Asp801Asn, p.Glu815Lys, p.Gly947Arg	*ATP1A3*: c.2443G > A (p.Glu815Lys)	p.Glu815Lys associated with severe phenotypes in both
KD response	3/10 responders: hemiplegia cessation (2 patients), reduced attack frequency/severity	Marked efficacy: hemiplegia frequency ↓70%, duration ↓50% Neurodevelopmental improvement	KD consistently reduced hemiplegia; limited effect on epilepsy
Neurodevelopmental outcomes	Moderate–severe ID: 9/10	Pre-KD: global delay, post-KD: improved Gesell scores	KD may mitigate neurodevelopmental impairment

AHC = alternating hemiplegia of childhood, ID = intellectual disability, KD = ketogenic diet.

The precise mechanism by which the KD treats AHC remains uncertain. Hunanyan AS^[14]^ introduced a new knockin mouse model (Mashl^±^ mouse) that carries the most prevalent heterozygous mutation (p.Asp801Asn) responsible for AHC. The CA1 pyramidal neurons in Mashl^±^ mice exhibit normal excitability when subjected to low frequency stimulation, the excitability increased by higher frequency single-pulse and paired-pulse stimulations. *ATP1A3* dysfunction is most likely to occur during periods of energy crisis and increased cell activity. Many symptoms of AHC manifest under conditions of enhanced excitability and stress, which may be associated with heightened neuronal cell activity, neuronal depolarization, and increased extracellular K^+^. *ATP1A3* pumps are responsible for transporting extracellular K^+^ to neurons, and Mashl^±^ mice are more susceptible to K^+^-induced spreading depression. Therefore, spreading depression could be a potential mechanism for seizures and hemiplegia observed in AHC. Oliveira M^[15]^ have demonstrated that a KD consisting of long-chain triglycerides or medium chain triglycerides can have a positive effect on cortical spreading depression in young rats when administered for a short duration. Based on the results of animal experimental studies, it is possible that the KD may alleviate the clinical symptoms of AHC patients by reducing the cerebral cortex’s spreading inhibition.

## 4. Conclusion

The KD may be an effective measure for the treatment of AHC, but the current sample size is small. Larger samples and more evidence-based data are needed to clarify the efficacy, safety, and tolerability of the diet in the treatment of AHC.

## Author contributions

**Conceptualization:** Yu Yang, Shuxiang Hu.

**Data curation:** Yu Yang, Yanru Huang.

**Formal analysis:** Yu Yang, Penghui Liu, Pei Li.

**Funding acquisition:** Shuxiang Hu.

**Investigation:** Yu Yang, Pei Li.

**Methodology:** Yu Yang, Penghui Liu, Pei Li, Yanru Huang.

**Project administration:** Yanru Huang.

**Software:** Penghui Liu.

**Supervision:** Shuxiang Hu.

**Writing – original draft:** Yu Yang, Penghui Liu.

**Writing – review & editing:** Shuxiang Hu.
